# Cumulative dose assessment with transformer‐based deformable image registration addition for cervical cancer patients

**DOI:** 10.1002/acm2.70135

**Published:** 2025-07-13

**Authors:** Dongdong Zhou, Yuheng Shao, Fuying Wan, Jiayi Chen, Yumeng Zhang, Jiaqi Feng, Yunfei Ye, Jinglan Zhou, Fubin Zeng, Qi Chen, Shaobin Wang, Heqing Lu, Liang Yang

**Affiliations:** ^1^ Department of Shanghai Key Laboratory of Maternal Fetal Medicine Shanghai Institute of Maternal‐Fetal Medicine and Gynecologic Oncology Shanghai First Maternity and Infant Hospital School of Medicine Tongji University Shanghai China; ^2^ Department of Nantong University Xinglin College Nantong China; ^3^ Department of School of Health Science and Engineering University of Shanghai for Science and Technology Shanghai China; ^4^ Department of MedMind Technology Co., Ltd. Beijing China

**Keywords:** cervical cancer, cumulative dose assessment, deformable image registration

## Abstract

**Purpose:**

Based on the combination mode of internal and external radiotherapy for cervical cancer, this study aimed to investigate an accurate transformer‐based deformable image registration (DIR) method for cumulative dose assessment.

**Methods and Materials:**

According to a retrospective analysis conducted on 180 patients with cervical cancer who underwent intracavitary brachytherapy (ICBT) and external beam radiation therapy (EBRT), this study proposed a mix‐transformer structure‐based deformable image registration (MTDIR) network for registering CT scans of ICBT and EBRT, followed by dose accumulation and assessment. The mean dice similarity coefficient (DSC) and Hausdorff distance (HD) of the rectum and bladder were computed to compare the performance of MTDIR with that of the state‐of‐the‐art VoxelMorph method and the DIR method provided by velocity. Additionally, the cumulative dose of the bladder and rectum from four methods was calculated: direct DVH parameter addition (DA), DIR‐based dose addition provided by Velocity (VA), VoxelMorph‐based dose addition (VoA), and MTDIR‐based dose addition (MA).

**Results:**

The mean DSC values of MTDIR, VoxelMorph, and Velocity for the bladder were 0.78, 0.75, and 0.72 for the registration between CT scans of EBRT and the last ICBT, respectively. The mean DSC values of the rectum were also equal to 0.58, 0.56, and 0.52. The mean HDmean values of MTDIR, VoxelMorph, and Velocity for the bladder were 4.81, 5.16, and 5.43, and the mean HDmean values of the rectum were 5.41, 5.81, and 6.93, respectively. For the registration between CT scans of ICBT, the mean DSC values of MTDIR, VoxelMorph, and Velocity for the bladder were obtained 0.82, 0.80, and 0.77, and the mean DSC values for the rectum were equal to 0.70, 0.68, and 0.63, respectively. The mean HDmean values of MTDIR, VoxelMorph, and Velocity for the bladder were 4.22, 4.58, and 4.79, and the mean HDmean values of the rectum were obtained 4.86, 5.17, and 5.64, respectively. The results generally suggested that MTDIR outperformed VoxelMorph and Velocity.

**Conclusions:**

The study findings demonstrated that the model developed based on parameters obtained from the proposed method exhibited higher registration accuracy.

## 1 INTRODUCTION

Cervical cancer represents the most prevalent cancer and also the primary cause of cancer‐related mortality among women in numerous countries.^1^ Combined radiotherapy, which typically includes external beam radiotherapy (EBRT) and intracavitary brachytherapy (ICBT), is essential in the management of cervical cancer.^2^ The combination of EBRT and ICBT is widely utilized for the treatment of locally advanced cervical cancer.^3^, ^4^ According to the 2020 NCCN guidelines and EMBRACE studies,^5^ patients with cervical cancer who undergo radical radiotherapy require a reasonable combination of internal and external irradiation.^6^

The cumulative doses of ICBT and external beam radiation therapy (EBRT) are necessary for calculating their combined dose. However, it is difficult to accurately assess the cumulative dose for the same patient because of differences in treatment posture (the internal irradiation for cervical cancer is in the lithotomy position, while the external irradiation is in the supine position), fractionation schedule (the internal irradiation dose fractionation is 6 Gy per fraction, whereas the conventional dose fractionation is 1.8 Gy per fraction for external irradiation), and other significant differences between EBRT and ICBT during treatment (such as tumor regression and bladder filling).^7^ Moreover, the geometric deformation of the applicator during brachytherapy may disrupt the originally designed internal irradiation dose distribution, making it more difficult to accurately achieve a reasonable superimposition of the internal and external irradiation doses. The clinical guidelines from the European Society for Radiation Therapy and Oncology (GYNGEC‐ESTRO) suggest using the direct dose volume histogram (DVH) parameter accumulation method for evaluating the cumulative dose of EBRT and ICBT. However, this method does not consider the three‐dimensional structure of organs, organ deformation, and the non‐uniformity of dose distribution. Consequently, it is not possible to accurately assess the radiation damage to normal organs with the direct DVH parameter addition method.^8^ The deformable image registration (DIR)‐based dose addition method is an effective solution to the problem of dose addition in ERBT and ICBT.^9^ This method involves the accumulation of DVH parameters for EBRT and each ICBT session utilizing the derived deformation vector field.^10^ The Velocity imaging data management system (Varian Medical Systems; Palo Alto, USA) is currently the predominant commercial software for deformable registration, facilitating voxel correspondence between EBRT and ICBT images. However, the DIR model of Velocity is based on the B‐Spline method, which requires a careful control point setting strategy.^11^ The control point setting strategy may result in unregistered object details. Conversely, an uneven arrangement of control points, such as those determined by the object contour, may yield satisfactory registration solely in areas adjacent to the control points. Therefore, Velocity may fail to achieve accurate deformable registration in clinical scenarios due to the complex spatial correspondence between different image sets (between EBRT and ICBT or between different ICBT sessions). Several studies have shown significant differences between direct addition and Velocity addition.^12^, ^13^, ^14^ Kadoya et al. demonstrated that the calculated dose of OAR using Velocity addition tended to be overestimated^12^ compared to the direct DVH parameter addition method.

Recent advancements have been made in several deep learning‐based DIR methods.^15^, ^16^, ^17^, ^18^ Specifically, VoxelMorph^15^ was proposed to directly generate the deformation field between the input image pair via a Convolutional Neural Network (CNN). These methods, however, do not incorporate voxel‐level correlation modeling between the reference image and the moving image.^15^, ^16^, ^17^ Subsequently, methods based on transformer architecture gained attention within the research community due to the self‐attention mechanism.^19^ An attention mechanism maps a query vector to a set of key‐value pairs, producing an output vector. The query, keys, values, and output are all elements of the vector space. Initially, a weight is calculated for each value to obtain the output. The weight is established through the application of a compatibility function that utilizes the query and the associated key as inputs. The compatibility function quantifies the degree of alignment between the query and each key. Once the weights are computed, the output is generated by taking a weighted sum of the values. Each value is multiplied by its corresponding weight, and then these weighted values are summed up to produce the final output vector. This process allows the attention function to selectively focus on different values based on their relevance to the query. However, the large model size and GPU consumption requirements of previous transformer‐based methods^17^, ^18^ hinder their practical application. To address these limitations, this study proposes the mix‐transformer structure‐based deformable image registration (MTDIR) network. This network aims to align each voxel of the reference image with its corresponding voxel in the moving image by explicitly modeling voxel‐level correlations using a transformer‐based mix‐attention architecture.^19^, ^20^ Accordingly, the area in the input space that affects the output of the neuron encompasses the entire input image. In other words, this network provides a global receptive field. The MTDIR network also utilizes a global receptive field to achieve a more precise voxel‐level deformation vector field for the computation of cumulative dose distribution.

## 2 METHODS AND MATERIALS

### 2.1 Patients characteristics

This was a retrospective single‐institution study that collected and analyzed clinical data and CT scans of 180 patients who received internal and external radiation in the research team's hospital. The study was approved by the Ethics Committee of the hospital. Both the CT scans of ICBT and external irradiation were scanned using the GE Discovery CT590RT (General Electric Company, Fairfield, USA). The slice thickness of CT scans was 5 mm for EBRT and 2.5 mm for ICBT. The CT scan mode was Metal Artifact Reduction (MAR mode). A total of 180 patients were divided into a training‐validation dataset (n=100) and a testing dataset (n=80). Table 1 summarizes the clinical information of the participants. The participants aged from 32 to 80 years, with a median of 58 years. All participants were followed up for 6 to 50 months, with a median follow‐up period of 28 months. Clinical staging was determined according to the International Federation of Gynecology and Obstetrics (FIGO) classification for cervical cancer (2018). The standard chemotherapy regimen at the studied center during radiation therapy involved administering cisplatin at a dosage of 30 ${\mathrm{mg}/m}^{2}$ body surface area every week.

**TABLE 1:** Patient characteristics.

Age	Count and frequency (%)
20–44	24 (13.33)
45–64	104 (57.78)
65–80	52 (28.89)
**FIGO stage**	**Count and frequency (%)**
IB	18 (10.00)
IIA	10 (5.56)
IIB	36 (20.00)
IIIA	8 (4.44)
IIIB	5 (2.78)
IIIC	83 (46.11)
IVA	7 (3.89)
IVB	13 (7.22)
**Pathology**	**Count and frequency (%)**
Squamous cell carcinoma (SqCC)	148 (82.22)
Adenocarcinoma (Adeno)	11 (6.11)
Endometrioid (Endo)	21 (11.67)
**Number of chemotherapy treatments**	**Count and frequency (%)**
none	16 (8.89)
<5	81 (45.00)
≥5	83 (46.11)
**Radiotherapy external beam fraction**	**Count and frequency (%)**
45/25	15 (8.33)
48.6/27	18 (10.00)
50.4/28	147 (81.67)
**Boost to lymph node or Parametrium**	**Count and Frequency (%)**
None	117 (65.00)
56–58.8	54 (30.00)
58.8–61.6	9 (5.00)
**Applicator**	**Count and frequency (%)**
Cylindrical applicator	72 (40.00)
Intrauterine applicator combined with vaginal ovoids	103 (57.22)
Intrauterine applicator combined with interstitial needles	5 (2.78)
**ICBT fractionation**	**Count and frequency (%)**
12 Gy/2f	7 (3.89)
18 Gy/3f	63 (35.00)
24 Gy/4f	9 (5.00)
30 Gy/5f	88 (48.89)
36 Gy/6f	4 (2.22)
28 Gy/4f	9 (5.00)

### 2.2 EBRT

Dose calculations for EBRT treatment planning were conducted utilizing the Anisotropic Analytical Algorithm within Eclipse version 15.6 (Varian Medical Systems; Palo Alto, USA). The radiotherapy plan was developed using IMRT technology with nine evenly divided fields. The AAA algorithm was utilized, ensuring that 95% of the prescription dose encompassed 99% of the Planning Target Volume (PTV), while 100% of the prescription package covered at least 95% of the PTV volume. The radiation dose to the bladder and rectum was constrained to ensure that $D_{1cc}$ remained below 52 Gy and $V_{45}$ was less than 50%. The participants were required to have a bowel movement before positioning. They were instructed to drink 600–800 mL of water to measure bladder volume using B‐mode ultrasonography. CT scanning was initiated when the bladder volume exceeded 150 mL, based on the participant's tolerance level. The same intestinal preparation was performed before each treatment.

### 2.3 ICBT

The ICBT system employed the Brachy Vision 15.5 system (Varian Medical Systems; Palo Alto, USA) for dose calculation. Remote afterloader (GammaMedplus iX) was also used for brachytherapy. The cylindrical applicator mainly uses the Universal Stump applicator provided by Varian, and the intrauterine applicator combined with vaginal ovoids mainly employs the Manchester applicator provided by Varian. The interstitial needles were of reusable stainless steel needles manufactured by Varian. The CT images used for dose calculation were obtained from the GE Discovery CT590 RT. Before the treatment, the participants were instructed to empty their rectum and bladder. According to the specific conditions of each participant, a certain amount of normal saline was injected into the bladder through a urinary catheter before the positioning scan. Once the positioning was finished, the bladder was emptied. Subsequently, an equivalent volume of normal saline, matching that used during positioning, was reintroduced into the bladder before the treatment to maintain a consistent urine volume within the bladder. The ICBT plan necessitated that $D_{90}$ surpass the prescribed dosage, while the dose limit for rectum $D_{2cc}$ was established at below 80 Gy in terms of the 2 Gy Equivalent dose (EQD2). Similarly, the bladder $D_{2cc}$ was limited to less than 90 Gy in terms of the EQD2.

### 2.4 Framework of DIR based dose addition and assessment

**FIGURE 1:**
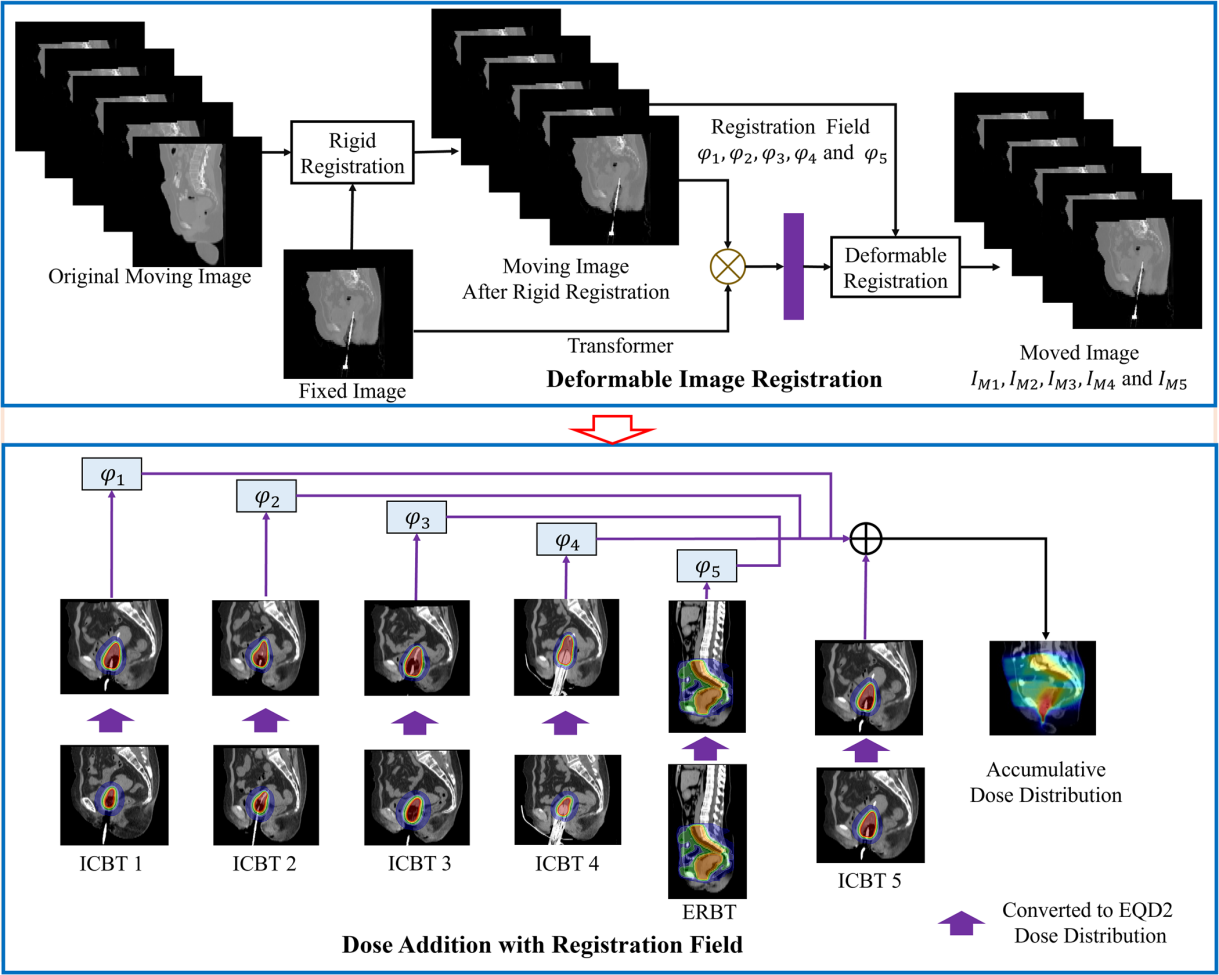
An overview of the proposed dose addition framework based on (MTDIR) in the combined radiotherapy of cervical cancer. DVH, dose volume histogram; EQD2, equivalent dose; ICBT, intracavitary brachytherapy; MTDIR, mix‐transformer structure‐based deformable image registration.

Figure 1 presents the complete process of the proposed methodology. This method employed the ICBT image from the previous session as the fixed image, while the ERBT image and additional ICBT images from various sessions served as the moving images. The fixed image and each moving image were inserted into the mix‐transformer‐based deformable registration network to obtain an accurate deformation field. This deformation field captures voxel‐level correspondence between the input image sets. The computed deformation field facilitated the accumulation of doses from various ICBT sessions and ERBT. Before accumulating all dose volumes, EBRT and ICBT doses were converted into EQD2 on a voxel‐by‐voxel level using linear‐quadratic model‐based equations by setting $\frac{\alpha }{\beta }$ to 3 Gy.^21^ Subsequently, specific DVH parameters such as $D_{2cc}$, $D_{0.1cc}$ and $V_{50}$ were also computed.

### 2.5 Details of the proposed deformable image registration network

#### 2.5.1 Network architecture

**FIGURE 2:**
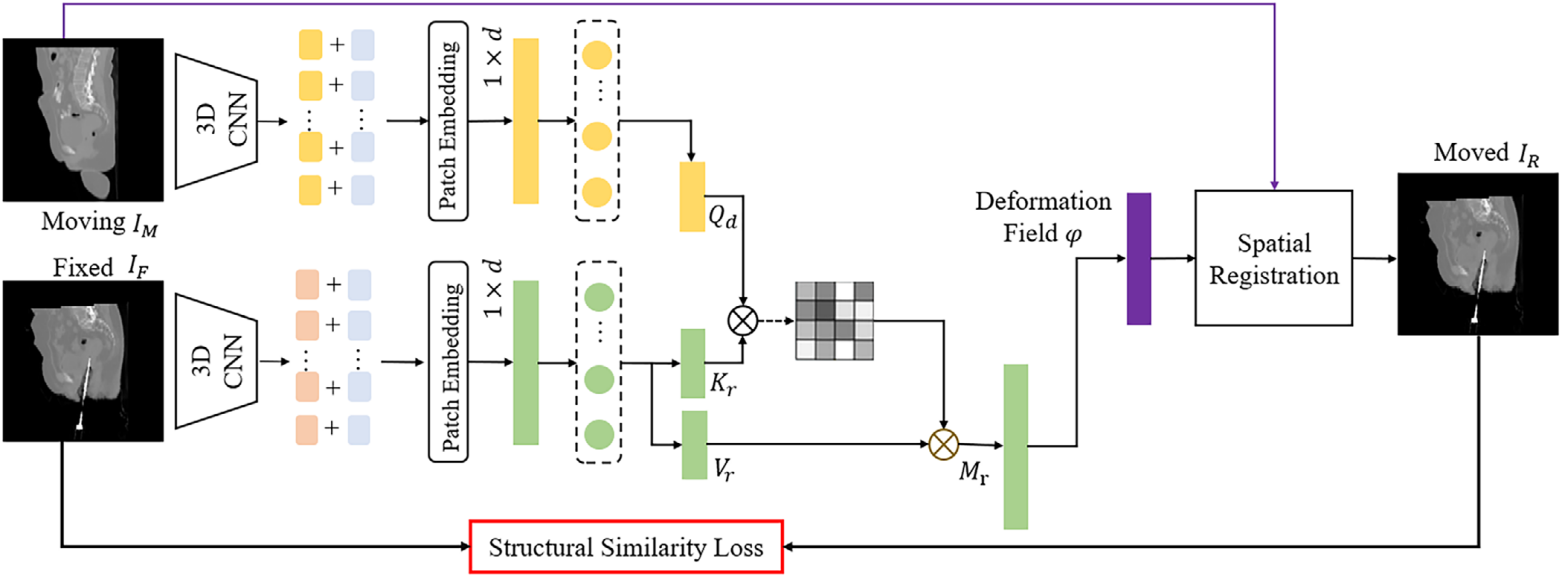
The detailed architecture of the MTDIR network. MTDIR, mix‐transformer structure‐based deformable image registration.

Figure 2 shows that the moving image $I_{M}$ and the fixed image $I_{F}$ were first processed by two 3D CNNs, resulting in the generation of $F_{M}$ and $F_{F}$, both in 3D form. The spatial resolution along the width and height dimensions was reduced by a factor of 16. To facilitate the subsequent processing using the transformer architecture, the generated 3D features $F_{M}$ and $F_{F}$ were reshaped into one‐dimensional form. They were then fused with positional embedding through element‐wise summation. This enabled the extraction of patch embedding vectors, which were further transformed into a dimension of (1×d).

In the given scenario, the feature embedding of the moving image served as the query vector $Q_{d}$ in the transformer correlation modeling, and the feature embedding of the reference image served as the key vector $K_{r}$ and the value vector $V_{r}$. The mix‐attention mechanism was then applied to model the correlation between the features of the moving and reference images as follows:

(1) $$M_{r}=Softmax\left(\frac{Q_{d}{K_{r}}^{T}}{\sqrt{dim(V_{r})}}\right)V_{r}$$

The formula illustrates that the query vector $Q_{d}$ interacts with the transposed key vector $K_{r}$ through multiplication, resulting in the similarity coefficient (attention score) between the query sequence and the key sequence. To enhance calculation stability, the generated attention score is typically divided by a scaling factor, specifically the square root of the value vector's length, resulting in a scaled attention score. Then, the scaled score is normalized through the softmax function to obtain the attention weight. Finally, a weighted sum of the value sequence is performed according to the attention weight matrix to obtain the cross‐attention representation, generating the correlation features. The generated correlation features $M_{r}$ are then inputted into a decoder that consists of a 3D CNN. This decoder is responsible for predicting the deformation field. Once the deformation field is predicted, it is utilized to spatially transform the moving image, resulting in the registered image $I_{R}$. To ensure the quality of the registration, the learning process involves a loss function that enforces structural similarity between the reference image $I_{F}$ and the registered image $I_{R}$. This loss function quantifies the similarity in structural patterns, textures, or other visual attributes between the two images. Optimizing this loss function enables the model to generate precise deformations and attain improved alignment between the reference and registered images.

**FIGURE 3:**
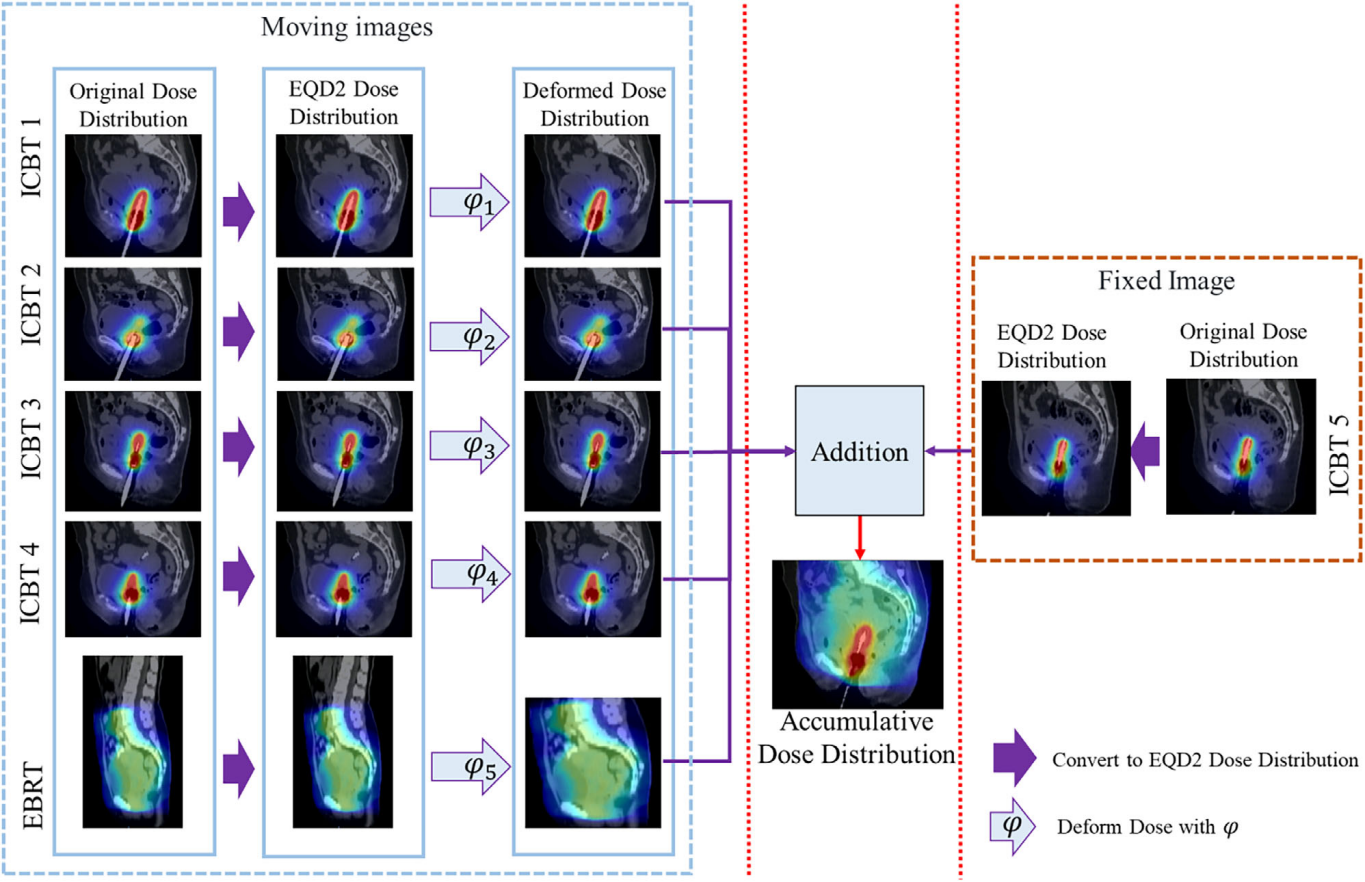
The framework of calculation of cumulative dose using mix‐transformer structure‐based deformable image registration (MTDIR). First, the original dose distribution is converted to an EQD2. The EQD2 distribution is then deformed based on the deformation field obtained from MTDIR. The target image ICBT5 is overlaid, and the cumulative dose distribution is calculated. EBRT, external beam radiation therapy; EQD2, equivalent dose in 2 Gy fractions; ICBT, intracavitary brachytherapy; MTDIR, mix‐transformer structure‐based deformable image registration.

The deformation field obtained from the proposed MTDIR network allows for the calculation of the cumulative dose distribution of EBRT and ICBT. Figure 3 demonstrates the overall framework of cumulative dose calculation using the learned deformation field. The EBRT and all ICBT doses must initially be converted to EQD2 through a linear‐quadratic model‐based transformation. The proposed MTDIR deformable registration network is then used to compute the deformation field, facilitating the alignment of the dose distribution from the moving image with that of the fixed image. The use of a consistent coordinate system allows for the cumulative assessment of dose distributions from ICBT and EBRT, leading to total doses for organs at risk (OARs). The computed deformation field is also used to transform the dose distributions of ICBT sessions and EBRT into a unified coordinate system, allowing for the calculation of the cumulative dose distribution. DVH parameters are then computed based on the cumulative dose distribution. Accordingly, it is possible to quantitatively evaluate the benefit of accurate DIR for cumulative dose assessment.

### 2.6 Loss function

To supervise the learning of the proposed MTDIR network, the loss function consists of two parts:

1. Image similarity loss.
  The image similarity loss is used to enforce the similarity between the moving image ($I_{M}$) deformed with the deformation field ($I_{M}\circ \phi$) and the reference image ($I_{F}$). The local normalized cross‐correlation loss^15^ is employed to guide the learning of our DIR model:
(2) $$\begin{array}{cc} L_{\text{sim}} & =L_{\text{NCC}}\left(I_{F},I_{M},\phi \right)\\ & =\sum_{p\in \Omega }\frac{{\left(\sum_{p_{i}}\left(I_{F}\left(p_{i}\right)-\bar{I_{F}}\left(p\right)\right)\left(\left[I_{M}\circ \phi \right]\left(p_{i}\right)-\left[\bar{I_{M}}\circ \phi \right]\left(p\right)\right)\right)}^{2}}{\left(\sum_{p_{i}}{\left(I_{F}\left(p_{i}\right)-\bar{I_{F}}\left(p\right)\right)}^{2}\right)\left(\sum_{p_{i}}{\left(\left[I_{M}\circ \phi \right]\left(p_{i}\right)-\left[\bar{I_{M}}\circ \phi \right]\left(p\right)\right)}^{2}\right)} \end{array}$$
  where ${\bar{I}}_{F}$ and ${\bar{I}}_{M}\circ \phi$ represent the images with local mean intensities subtracted out: ${\bar{I}}_{F}=I_{F}\left(p\right)-\frac{1}{n^{3}}\sum_{p_{i}}I_{F}\left(p_{i}\right)$, where $p_{i}$ iterates over a $n^{3}$ volume around p. Following ref. [15], we set n=9
2. Deformation field regularization loss.
  To effectively supervise the learning of the proposed model, reliance solely on the aforementioned image similarity loss may result in a deformation field that lacks smoothness. The inclusion of a smoothness constraint necessitates the addition of a deformation field regularization loss. The deformation field regularization loss promotes a smoother learned deformation field by encouraging smaller values. Specifically, the following diffusion regularizer is employed:
(3) $$L_{smmoth}=R_{diffusion}\left(\varphi \right)=\sum_{p\in \Omega }{\left||\nabla u\left(p\right)|\right|}^{2}$$
  In this equation, ∇u(p) is the spatial gradient of the deformation field. The overall loss function can be formulated as follows:
(4) $$L\left(I_{f},I_{m},\phi \right)=L_{\text{sim}}\left(I_{F},I_{M},\phi \right)+\lambda L_{\text{smooth}}$$
  where λ and γ are the weighting factors, which control the strength of deformation field regularization loss term $L_{\text{smooth}}$.

### 2.7 Model training details

The testing set consisted of 80 participants, while the training‐validation set included 100 participants (Table 1). A five‐fold cross‐validation strategy was employed to select the model with the highest validation performance. The NVIDIA A6000 GPU was used as the training device, with a learning rate of 0.0001 and a maximum of 400 epochs.

### 2.8 Performance evaluation

#### 2.8.1 DSC and Hausdorff distance of bladder and rectum

Based on previous studies, the accuracy of MTDIR was evaluated using the dice similarity coefficient (DSC),^22^, ^23^ and Hausdorff distance (HD). The DSC measures the similarity score between two sets and is commonly used to estimate the similarity between the reference mask (A) and deformed mask (B); it is formulated as follows:

(5) $$\text{DSC}=\frac{2(A\cap B)}{A+B}$$

This study computed the 3D DSC value between the deformed and original images. The DSC value varies between 0 and 1, with 0 representing no overlap and 1 signifying complete overlap. HD also measures the similarity between two boundary point sets (A and B) of the generated mask.

(6) $$\begin{array}{cc} \text{HD}(A,B) & =\max\left(h(A,B),h(B,A)\right)\\ h(A,B) & =\max_{a\in A}\left\{\min_{b\in B}∥a-b∥\right\} \end{array}$$

Specifically, HD95 is based on the 95^th^ percentile of the distances between boundary points in two sets. Similarly, HDmean computes the similarity with the mean of the distances between boundary points in two sets. Lower HD95 and HDmean values indicate improved overlap between the two sets. The mean DSC score and HD value for all participants in the testing set were calculated using the masks of the bladder and rectum from each ICBT and ERBT image. For each participant, the mask of the final ICBT image served as the reference. The masks of the other ICBT images and the ERBT image were altered utilizing the calculated deformation field. Then DSC scores and HD values between the deformed masks and the reference mask were calculated.

The proposed MTDIR method was compared against the state‐of‐the‐art methods, that is, VoxelMorph and Velocity, which served as robust baselines. Moreover, the extended deformable multi‐pass (EXDMP) method was chosen for performing DIR using Velocity.

#### 2.8.2 Statistical analysis

The Friedman test was employed for multiple comparisons of the DVH parameters derived from four different methods. Then post hoc tests were conducted for pairwise comparisons. The Bonferroni method was utilized to adjust the significance level. In addition, the paired Wilcoxon rank‐sum test was employed to compare the DSC between MTDIR and state‐of‐the‐art methods. The statistical significance was set at a two‐tailed P‐value of less than 0.05. The Bonferroni correction method indicated that the statistical significance level among the four methods was equal to 0.0125.

## 3 RESULTS

### 3.1 Comparison of image registration methods

**FIGURE 4:**
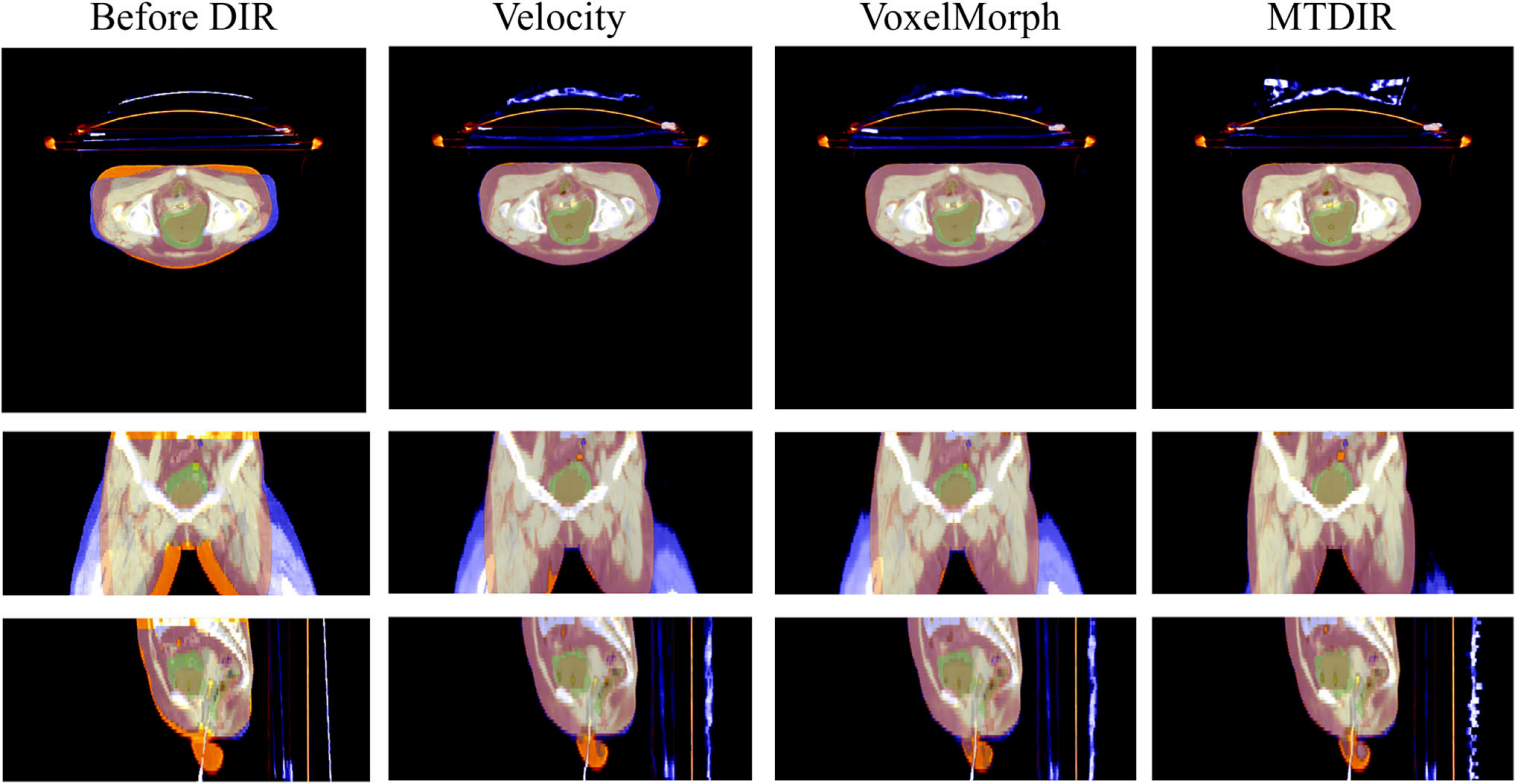
Visualized comparison of the registration performance between Velocity, VoxelMorph and the proposed MTDIR. MTDIR, mix‐transformer structure‐based deformable image registration.

The mean DSC scores for the original mask of the fixed image and the moving images were computed before the application of DIR (Table 2). The mean DSC scores between EBRT and the last ICBT for the bladder and the rectum were 0.65 and 0.48. For the registration between the last ICBT and other ICBTs, the mean DSC scores were 0.69 for the bladder and 0.56 for the rectum. Moreover, the mean HD95 values between EBRT and the last ICBT for the bladder and rectum were 9.42 and 10.79, and the mean HD95 values between the last ICBT and other ICBTs were 8.55 and 9.34. Finally, the mean HDmean values for the bladder and the rectum were 6.85 and 7.97 between CT scans of EBRT and the last ICBT and 5.72 and 6.95 between CT scans of ICBT. Subsequently, the mean DSC scores, HD95, and HDmean values were calculated after the application of the DIR transformation. The proposed MTDIR demonstrated mean DSC scores of 0.78 and 0.58 for the bladder and rectum, respectively, in the registrations between the last ICBT and EBRT. These results indicate that the proposed MTDIR outperformed VoxelMorph, which achieved scores of 0.75 and 0.56, and Velocity, which recorded scores of 0.72 and 0.52. MTDIR demonstrated mean HD95 values of 7.26 and 7.07 for the bladder and rectum, respectively, outperforming VoxelMorph, which recorded values of 7.89 and 8.54. MTDIR also achieved the highest results for the mean HDmean of the bladder and rectum, with values of 4.81 and 5.41, which are better compared to those of Velocity (5.43 and 6.93) and VoxelMorph (5.16 and 5.81). Furthermore, the proposed MTDIR demonstrated mean DSC scores of 0.82 for the bladder and 0.70 for the rectum in registrations between the last ICBT and other ICBTs, surpassing VoxelMorph's scores of 0.80 and 0.68, as well as Velocity's scores of 0.77 and 0.63. Similarly, the HD95 for the proposed MTDIR yielded results of 6.10 and 6.46 for the bladder and rectum, respectively, exceeding the VoxelMorph results of 6.75 and 6.92. MTDIR outperformed Velocity (4.79 and 5.64) and VoxelMorph (4.58 and 5.17) in terms of HDmean, achieving the best results for the bladder and rectum (4.22 and 4.86). Figure 4 shows the visualized registration results between the ICBT and EBRT of Velocity, VoxelMorph, and the proposed MTDIR for one participant from the testing set to provide a clear understanding of the superiority of the proposed MTDIR method. The masks of the bladder and rectum are also presented for enhanced comprehension. The visualized comparison indicates that the proposed MTDIR achieves registration results that are more closely aligned with the EBRT.

**TABLE 2:** A comparison between the image registration methods in the DIR accuracy.

Method	Bladder	Bladder	Bladder	Rectum	Rectum	Rectum
	(DSC ± std.)	(HD95 ± std.)	(HDmean ± std.)	(DSC ± std.)	(HD95 ± std.)	(HDmean ± std.)
Registration between the last ICBT and EBRT						
Before DIR	0.65 ± 0.14	9.42 ± 2.55	6.85 ± 2.86	0.48 ± 0.19	10.79 ± 3.61	7.97 ± 4.15
Velocity	0.72 ± 0.15	/	5.43 ± 2.68	0.52 ± 0.19	/	6.93 ± 3.72
VoxelMorph	0.75 ± 0.15	7.89 ± 1.68	5.16 ± 1.97	0.56 ± 0.18	8.54 ± 2.67	5.81 ± 1.87
MTDIR	**0.78 ± 0.16**	**7.26 ± 1.63**	**4.81 ± 1.82**	**0.58 ± 0.19**	**7.07 ± 2.59**	**5.41 ± 1.78**
Chi‐square	13.62	8.96	14.70	10.84	10.25	14.76
*p*‐value	0.002	0.007	0.002	0.013	0.007	0.002
Registration between the last ICBT and other ICBTs						
Before DIR	0.69 ± 0.15	8.55 ± 1.40	5.72 ± 2.38	0.56 ± 0.19	9.34 ± 3.34	6.95 ± 2.76
Velocity	0.77 ± 0.16	/	4.79 ± 2.10	0.63 ± 0.18	/	5.64 ± 2.42
VoxelMorph	0.80 ± 0.16	6.75 ± 1.47	4.58 ± 1.84	0.68 ± 0.18	6.92 ± 2.72	5.17 ± 1.93
MTDIR	**0.82 ± 0.16**	**6.10 ± 1.38**	**4.22 ± 1.72**	**0.70 ± 0.20**	**6.46 ± 2.63**	**4.86 ± 1.81**
Chi‐square	15.02	9.86	14.37	13.29	9.26	14.04
*p*‐value	0.002	0.007	0.002	0.004	0.007	0.003

Abbreviations: DIR, deformable image registration; DSC, dice similarity coefficient; EBRT, external beam radiation therapy; ICBT, intracavitary brachytherapy; MTDIR, mix‐transformer structure‐based deformable image registration.

### 3.2 Comparison of DVH addition methods

Table 3 shows the details of the mean DVH parameters for the rectum and bladder of all the participants in the testing set. The Friedman test results indicate a statistically significant difference in various dose accumulation methods for all DVH parameters of the bladder and for certain DVH parameters of the rectum. DA achieved the smallest DVH parameters for both the bladder and rectum. In terms of $D_{2cc},D_{1cc}$ and $D_{0.1cc}$, the VA obtained the largest DVH parameters for both the bladder and rectum. Moreover, the MA achieved the largest $V_{50}$ for the bladder and rectum.

**TABLE 3:** Comparison of DVH parameters with different DVH addition methods. ()

Method	$D_{2cc}$	$D_{1cc}$	$D_{0.1cc}$	$V_{50}$
**Bladder**				
DA	65.90 ± 7.66	68.27 ± 8.52	74.65 ± 10.92	24.31 ± 13.71
VA	72.82 ± 11.40	77.32 ± 13.76	92.86 ± 23.34	37.34 ± 13.21
VoA	68.27 ± 9.04	72.35 ± 10.79	83.51 ± 21.14	41.23 ± 8.49
MA	66.31 ± 8.97	69.17 ± 10.45	79.43 ± 20.19	47.47 ± 5.94
Chi‐square	6.734	8.033	9.985	40.678
*p*‐value	0.002	0.001	<0.001	<0.001
Variance*	DA‐VA (0.002)	DA‐VA (<0.001)	DA‐VA (<0.001)	DA‐VA (<0.001)
	DA‐VoA (0.713)	DA‐VoA (0.527)	DA‐VoA (0.098)	DA‐VoA (<0.001)
	DA‐MA (0.800)	DA‐MA (0.648)	DA‐MA (0.181)	DA‐MA (<0.001)
	VA‐VoA (0.009)	VA‐VoA (0.006)	VA‐VoA (0.012)	VA‐VoA (<0.001)
	VA‐MA (0.005)	VA‐MA (0.003)	VA‐MA (0.006)	VA‐MA (<0.001)
	VoA‐MA (0.004)	VoA‐MA (0.003)	VoA‐MA (0.004)	VoA‐MA (<0.001)
**Rectum**				
DA	67.04 ± 5.07	70.13 ± 5.83	77.28 ± 7.66	25.16 ± 16.65
VA	67.44 ± 10.74	72.92 ± 15.12	92.84 ± 32.55	47.75 ± 14.03
VoA	66.98 ± 8.12	71.17 ± 9.26	83.19 ± 12.65	48.06 ± 7.96
MA	66.31 ± 6.87	70.03 ± 7.87	78.86 ± 11.46	49.89 ± 6.30
Chi‐square	0.428	1.668	10.485	87.628
*p*‐value	0.650	0.191	<0.001	<0.001
Variance*	DA‐VA (0.842)	DA‐VA (0.178)	DA‐VA (0.178)	DA‐VA (<0.001)
	DA‐VoA (0.463)	DA‐VoA (0.285)	DA‐VoA (0.457)	DA‐VoA (<0.001)
	DA‐MA (0.349)	DA‐MA (0.768)	DA‐MA (0.768)	DA‐MA (<0.001)
	VA‐VoA (0.324)	VA‐VoA (0.217)	VA‐VoA (0.189)	VA‐VoA (0.098)
	VA‐MA (0.418)	VA‐MA (0.148)	VA‐MA (0.148)	VA‐MA (<0.001)
	VoA‐MA (0.125)	VoA‐MA (0.086)	VoA‐MA (0.077)	VoA‐MA (0.130)

*Note*: The Bonferroni corrected significance level was 0.0125.

Abbreviations: $D_{0.1cc}$, the highest dose received within 0.1 cubic centimeters; $D_{1cc}$, the highest dose received within 1 cubic centimeter; $D_{2cc}$, the highest dose received within 2 cubic centimeters; DA, direct dose addition; MA, MTDIR addition; VA, velocity addition; VoA, VoxelMorph addition.

* The variance measures the consistency of numerical metrics (D2cc, D1cc, D0.1cc and V50) between two methods. The smaller the variance, the more consistent quantitative metric between the two methods.

## 4 DISCUSSION

Accurate deformable registration is essential in cervical cancer radiotherapy for the integration of external and internal radiation therapy. This study proposed the MTDIR deformation registration algorithm for the superimposition of internal and external irradiation doses. The study findings suggested that MTDIR outperformed the leading methods, VoxelMorph and Velocity, in the deformable registration accuracy. This study employed the global receptive field of the Transformer and introduced a hybrid Transformer attention mechanism to learn the correlation between each voxel of the moving image and each voxel of the fixed image, thereby enhancing registration accuracy. Experimental results demonstrated that the proposed deformable registration method outperformed Velocity and VoxelMorph in terms of registration effectiveness. The DSC values for the bladder and rectum were similar to the results reported in reference.^13^ Teo et al. reported that the mean DSC values for the rectum and bladder were both greater than 0.90. A potential explanation for their superior results is the implementation of rigorous bowel preparation and image preprocessing, which mitigated the effects of artifacts like air and contrast agents on registration accuracy.^24^ This study identified differences in bowel preparation methods between external and internal radiation scans. The external radiation employed a method involving the consumption of water and urine retention, whereas the internal radiation utilized water injection via a urinary catheter. This resulted in notable discrepancies in bladder volumes between the external and internal radiation scans, with some participants receiving external radiation failing to fully evacuate their rectum. The presence of air in the rectum, exhibiting distinct Hounsfield Unit (HU) values compared to normal tissues, can significantly affect registration accuracy. Additionally, the different upper and lower boundary definitions for rectum delineation in the combined internal and external radiation may cause differences in the calculated DSC values. Currently, there is no consensus on clinically significant DSC values.^12^

There are many uncertainties in the dosimetric parameters obtained from deformable registration. Hayashi et al.^25^ studied the cumulative dose of external and internal radiation in the rectum in 14 patients using Varian Velocity AI software and found that the DVH method overestimated the doses of OAR $D_{0.1cc}$, $D_{1cc}$, and $D_{2cc}$ compared to the deformable registration method. Van Heerden et al.^24^, ^26^, ^27^ reported the same results, indicating that the high‐dose volume did not overlap because of the non‐uniform distribution of EBRT and BT. This suggests that the “worst‐case assumption” advised by GEC‐ESTRO for DVH overlay was not realized. However, Kim and Kadoya^12^, ^28^ found that the deformable registration method resulted in higher cumulative doses of OAR $D_{0.1cc}$, $D_{1cc}$, and $D_{2cc}$ compared to the DVH method, suggesting that inaccurate deformable registration could lead to errors in dose accumulation. Additionally, Zhao et al. and Abe^13^, ^29^ used MIM (MIM Software Inc.; Cleveland, OH, USA) and Velocity software, respectively, to study the differences between deformable registration dose accumulation and the DVH method in cervical cancer internal radiation. Both studies found no significant difference in the mean $D_{2cc}$ for the rectum and bladder. This study found that the mean $D_{0.1cc}$, $D_{1cc}$, and $D_{2cc}$ obtained through deformable registration were higher compared to those of the DVH method. Further investigation revealed notable outliers, particularly in the VA method, likely resulting from the applicator's deformation at the edges or interior of the bladder and rectum during the registration process.

When excluding these data, it was observed that only the mean dose of $D_{0.1cc}$ obtained through deformable registration was higher than that from the DVH method, while there was no statistically significant difference in the mean doses of $D_{1cc}$ and $D_{2cc}$. This finding supports prior research suggesting that the accuracy of deformable registration influences small doses, such as $D_{0.1cc}$,^25^ while $D_{2cc}$ remains comparatively stable. When the three deformable registration methods were compared, Velocity showed higher mean doses of $D_{0.1cc}$ and $D_{1cc}$ compared to MTDIR, potentially due to a greater number of outliers produced by Velocity.

Similar to any other research project, this study faced several limitations. Firstly, the accuracy and reliability of deformable registration need further improvement, particularly in managing applicators during the deformation process, which may introduce uncertainties. Secondly, the existing training models only rely on imaging features, which often exhibit high correlations. Additional factors, such as the inconsistency of the organ size of ICBT and EBRT of different patients, may influence the registration results. Finally, the standardization of sample size treatment still needs to be improved. Furthermore, previous studies^30^, ^31^ have demonstrated a close correlation between rectum toxicity and DVH parameters through statistical analysis. Future studies are recommended to explore the advantages of accurate DIR in predicting toxicity to the bladder and rectum.

## 5 CONCLUSION

The objective of combined radiotherapy for cervical cancer is to improve dose accumulation and assessment through the integration of a deep learning‐based DIR method. The study results demonstrated that the proposed MTDIR network achieved superior registration accuracy and performance for dose accumulation. Therefore, it is crucial to develop more accurate DIR methods in order to achieve enhanced dose accumulation and assessment performance.

## AUTHOR CONTRIBUTIONS

Dongdong Zhou, Yuheng Shao, Fuying Wan, Heqing Lu and Liang Yang pointed out the problem of current methods and provided new solution. Dongdong Zhou, Heqing Lu and Liang Yang designed the dataset constructing scheme. Jiayi Chen, Yumeng Zhang, Jiaqi Feng collected the dataset. Yunfei Ye, Jinglan Zhou, Fubin Zeng cleaned the dataset. Dongdong Zhou, Yuheng Shao, Fuying Wan, Yunfei Ye, Jinglan Zhou, Fubin Zeng and Qi Chen performed the experiments. Dongdong Zhou, Jiayi Chen, Yumeng Zhang, Jiaqi Feng, Liang Yang, Heqing Lu and Shaobin Wang evaluated the experimental results. Dongdong Zhou, Yuheng Shao, Fuying Wan wrote the first draft of the manuscript. All the authors revised the manuscript. All the authors contributed to the article and approved the submitted version. All the authors approve the final version to be published and agree to be accountable for all aspects of the work in ensuring that questions related to the accuracy or integrity of any part of the work are appropriately investigated and resolved.

## CONFLICT OF INTEREST STATEMENT

The authors declare that the research was conducted in the absence of any commercial or financial relationships that could be construed as a potential conflict of interest.
